# DBGC: Dimension-Based Generic Convolution Block for Object Recognition

**DOI:** 10.3390/s22051780

**Published:** 2022-02-24

**Authors:** Chirag Patel, Dulari Bhatt, Urvashi Sharma, Radhika Patel, Sharnil Pandya, Kirit Modi, Nagaraj Cholli, Akash Patel, Urvi Bhatt, Muhammad Ahmed Khan, Shubhankar Majumdar, Mohd Zuhair, Khushi Patel, Syed Aziz Shah, Hemant Ghayvat

**Affiliations:** 1 Department of Computer Engineering, Devang Patel Institute of Advance Technology and Research (DEPSTAR), Faculty of Technology and Engineering (FTE), CHARUSAT Campus, Charotar University of Science and Technology (CHARUSAT), Changa 388421, India; chiragpatel.dce@charusat.ac.in (C.P.); urvashichaudhari.dce@charusat.ac.in (U.S.); khushipatel.ce@charusat.ac.in (K.P.); 2 Parul University, Vadodara 382030, Gujarat, India; dulari.bos@gmail.com; 3 Department of Information Technology, Devang Patel Institute of Advance Technology and Research (DEPSTAR), Faculty of Technology and Engineering (FTE), CHARUSAT Campus, Charotar University of Science and Technology (CHARUSAT), Changa 388421, India; radhipatel999@gmail.com (R.P.); akashpatel.dit@charusat.ac.in (A.P.); urvibhatt.dcs@charusat.ac.in (U.B.); 4 Symbiosis Institute of Technology, Symbiosis International (Deemed) University, Pune 412115, India; sharnil.pandya@sitpune.edu.in; 5 Sankalchand Patel College of Engineering, Sankalchand Patel University, Visnagar 384315, India; kjmodi.fet@spu.ac.in; 6 Department of Information Science and Engineering, R. V. College of Engineering, Banglore 560059, India; nagaraj.cholli@rvce.edu.in; 7 DTU Health Tech Department of Health Technology, 247 99 Lyngby, Denmark; mahkh@dtu.dk; 8 Department of Electronics and Communication Engineering, National Institute of Technology, Bijni Complex, Laitumkhrah, Shillong 793003, Meghalaya, India; shub@nitm.ac.in; 9 Department of Computer Science and Engineering, Institute of Technology, Nirma University, Ahmedabad 382481, India; md.zuhair.cs@gmail.com; 10 Healthcare Technology and Innovation Theme, Faculty Research Centre for Intelligent Healthcare, Coventry University, Richard Crossman Building, Coventry CV1 5RW, UK; syed.shah@coventry.ac.uk or; 11 Computer Science Department, Faculty of Technology, Linnaeus University, P G Vejdes väg, 351 95 Växjö, Sweden

**Keywords:** CNN, separable convolution, DBGC, dimension-based kernels

## Abstract

The object recognition concept is being widely used a result of increasing CCTV surveillance and the need for automatic object or activity detection from images or video. Increases in the use of various sensor networks have also raised the need of lightweight process frameworks. Much research has been carried out in this area, but the research scope is colossal as it deals with open-ended problems such as being able to achieve high accuracy in little time using lightweight process frameworks. Convolution Neural Networks and their variants are widely used in various computer vision activities, but most of the architectures of CNN are application-specific. There is always a need for generic architectures with better performance. This paper introduces the Dimension-Based Generic Convolution Block (DBGC), which can be used with any CNN to make the architecture generic and provide a dimension-wise selection of various height, width, and depth kernels. This single unit which uses the separable convolution concept provides multiple combinations using various dimension-based kernels. This single unit can be used for height-based, width-based, or depth-based dimensions; the same unit can even be used for height and width, width and depth, and depth and height dimensions. It can also be used for combinations involving all three dimensions of height, width, and depth. The main novelty of DBGC lies in the dimension selector block included in the proposed architecture. Proposed unoptimized kernel dimensions reduce FLOPs by around one third and also reduce the accuracy by around one half; semi-optimized kernel dimensions yield almost the same or higher accuracy with half the FLOPs of the original architecture, while optimized kernel dimensions provide 5 to 6% higher accuracy with around a 10 M reduction in FLOPs.

## 1. Introduction

The Convolution Neural Network is a widely used deep learning architecture for computer vision tasks such as object detection, object segmentation, and object recognition [1]. The basic building layer of a CNN is its convolution layer. Much research has been carried out to modify the CNN for various purposes. Several inspirational concepts for the progress of CNN have been investigated, including alternative activation functions, regularization, parameter optimization, and architectural advancement [1]. Some of the related research being carried out is summarized in this section in Table 1.

The main contribution of this paper is in designing a dimension selector module named DBGC (Dimension-Based Generic Convolution Unit). This module can be added into any architecture to reduce numbers of FLOPs without affecting accuracy. Two main contributions of the research lie in developing semi-optimized kernel and optimized kernel methods. Such methods reduce the number of FLOPs while providing equal or greater accuracy.

Computer vision and image processing have applications in fields such as traffic surveillance [2,3], object detection and segmentation [4,5,6], autonomous cars [7], agriculture [8,9,10,11], healthcare [12,13,14,15], video surveillance systems [16,17], sports [18,19], NLP, and many other fields. An important task for any computer vision application is to extract correct features [20]. It is mentioned in paper [21] that fusion methods for extracting features can be used for better performance. In [1], it is mentioned that there are eight categories of various kinds of CNN architectures. The proposed DBGC architecture is inspired from ShuffleNetv2 [22], ESPNetv2 [23], DiCENet [24], and MobileNetv2 [25]. The following sections explain the basic architectures of each of these networks, and also states their merits and demerits.

### 1.1. ShuffleNetv2

ShuffleNetv2 examines the network’s computational complexity using direct measurements such as speed and memory access cost (besides FLOPs, which acts as an indirect metric). Direct measurements are also evaluated by the target platform. ShuffleNetv2 was introduced in the 2018 paper ShuffleNetv2: Practical Guidelines for Efficient CNN Architecture Design. The study [24] was co-authored by Ningning Ma, Xiangyu Zhang, Hai-Tao Zheng, and Jian Sun. FLOPs is the industry standard metric for evaluating a network’s calculating performance. However, according to a few studies, the FLOPs metric does not fully expose some underlying realities; networks with similar FLOPs might still have varying speeds owing to memory access costs, parallelism, target platforms, and other considerations. Since none of these are fully accounted for with FLOPs, they are neglected. ShuffleNetv2 addresses these issues by presenting four network modelling principles, as shown below:

1. When the number of input channels and output channels are in the same proportion (1:1), memory access costs are minimized.
2. Excessive group convolution raises the cost of memory access: the group number should not be too large, as this would raise the memory access cost.
3. Fragmentation of the network diminishes the degree of parallelism: fragmentation decreases the network’s efficiency in performing parallel calculations.
4. Element-by-element procedures are not insignificant: although element-wise operations have few FLOPs, they can significantly increase memory access time.

**Figure 1:**
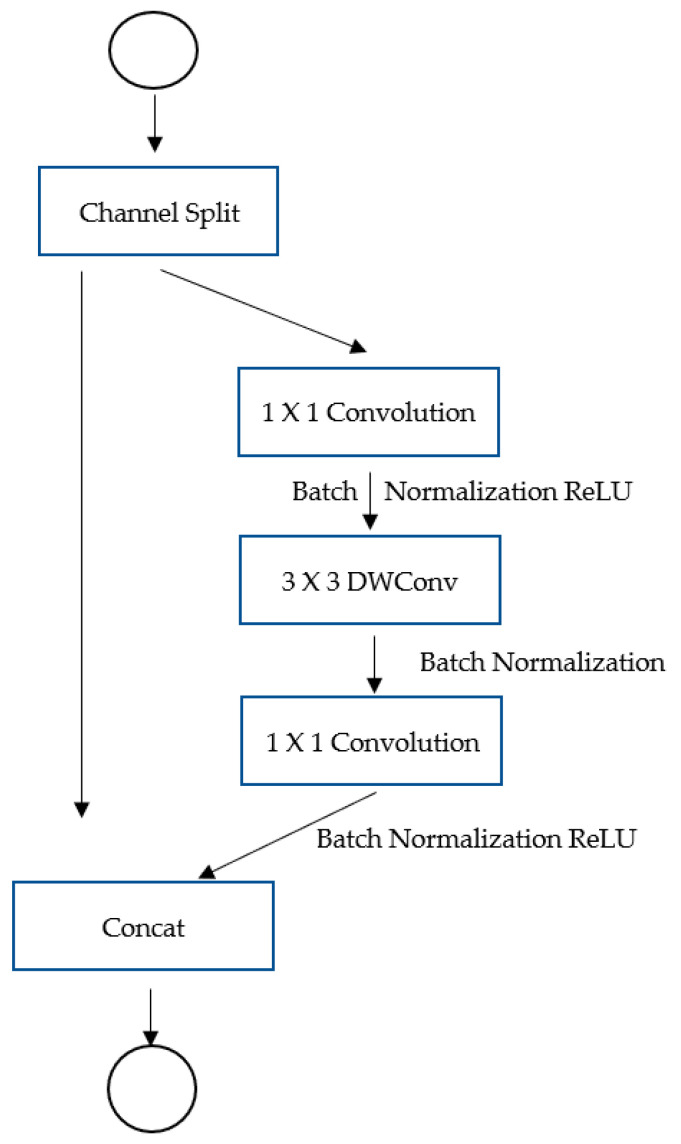
Design of ShuffleNetv2.

To boost network efficiency, all of these principles were incorporated into ShuffleNetv2 design as illustrated in Figure 1.

The channel split operator splits the channels into two groups, one of which is kept as the identity (third principle). Along the three convolutions, the other branch has an equal number of input and output channels (first principle). There are no group-wise convolutions in the 1 × 1 convolutions (second principle). ReLU concatenations and depth-wise convolutions are element-wise operations that are limited to a single branch (fourth principle).

### 1.2. ESPNetv2

**Figure 2:**
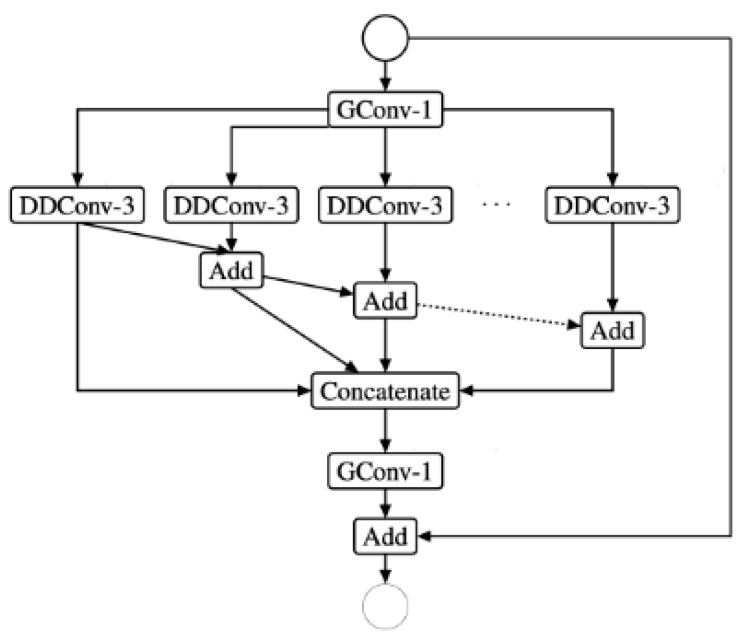
EESP building block.

ESPNetv1 was created with semantic segmentation in mind [26]. ESPNetv2 expands on the concepts of v1 and is designed for various computer vision tasks, including language modeling. The main goal is to separate the dilated convolutions depth-wise. EESP, which stands for Extremely Efficient Spatial Pyramid, is the new construction block shown in Figure 2.

ESPNetv1 employs group convolution for the 1 × 1 bottleneck layer (the first GConv-1 in the diagram), similarly to ShuffleNet, except that it does not perform channel shuffle. Instead, the dilated convolution is separated into the following two parts: first, a 3 × 3 depth-wise layer (DDConv-3), which is likewise dilated; and then an 11% point-wise layer. These concatenate results from the dilated layers and then apply a single 1 × 1 grouped convolution instead of computing K individual 1 × 1 layers (GConv-1 at the bottom). It is the same as the previous method, but it is more efficient.

PReLU is the activation function. The whole architecture begins with a stride of two regular convolution layers [27]. There are the following three phases after that: halving the spatial dimensions and doubling the number of channels; each stage beginning with a stride EESP block; the rest of the stage being built of EESP bricks in various shapes and sizes. After these three phases, there are a few additional convolution layers, global average pooling, and a fully-connected classifier layer. On dilated convolutions, the strided EESP has stride 2. Average pooling and concatenation replace the residual connection, doubling the number of chan·nels (doubling the number of filters in the convolution layers is slower). They also provide a long-range shortcut connection to the original picture. A depth-wise separable convolution follows one or more average pooling layers in this new connection. This long-range link should bring in some extra spatial information that would otherwise be lost as a result of down sampling. ESPNetv2 provides around 74% of Top1 accuracy for 5.9 M parameters.

### 1.3. DiCENet

The main idea of DiCENet was to replace regular convolution with dimension-wise convolution and fusion. As readers may be aware, depth-wise and group convolution slice the input tensor along the channel dimension, with each convolution filter operating on a subset of the channels. Why not cut along the width and height measurements as well? The disadvantage of depth-wise convolution is that it requires 11-layer convolutions to mix up the channels. All of these point-wise convolutions account for 90% of all operations in models such as MobileNet, according to DiCENet research. ShuffleNet attempted to address this by utilizing grouped convolution to speed up the 11 convolutions, but it may be possible to do better.

Convolution occurs across each dimension of the input data via a dimension-wise convolution, or “DimConv”. We may do convolution along three possible axes if the tensor is DHW: this is the well-known depth-wise convolution, abbreviated as HW. The filter window glides across the spatial dimensions (i.e., across the image’s width and height) while the input tensor is split along the channel axis. This is a width-wise convolution, which is DH. The filter window slides over an image of size DH as the input tensor is cut along the W axis. This is a height-wise convolution, denoted by DW. The filter window works on an image of size DW, and the input tensor is split along the H axis.

### 1.4. MobileNetv2

**Figure 3:**
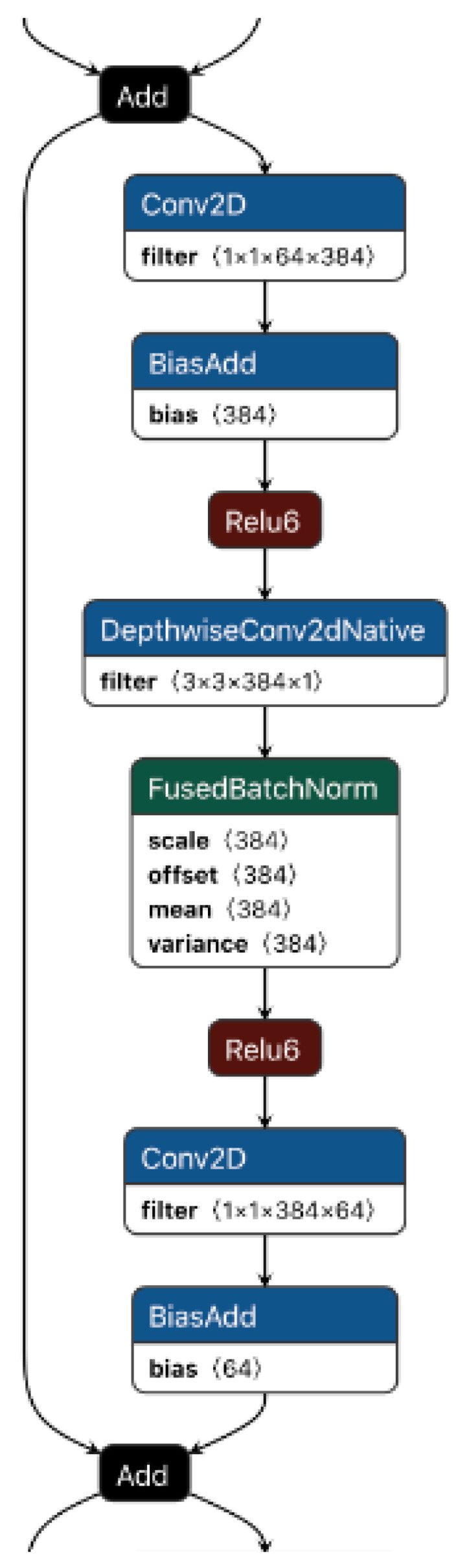
MobileNetv2.

MobileNetv2 is the same as MobileNetv1 and uses depth-wise convolution, but v2 rearranges the blocks as shown in Figure 3: the depth-wise convolution block is in the middle as per MobileNetv2 architecture. Before the depth-wise layer is a 1 × 1 convolution known as the expansion layer. This increases the number of channels. After the depth-wise layer is another 1 × 1 convolution that reduces the number of channels again, known as the projection layer or the bottleneck layer.

There is a residual connection when the number of channels entering into the block equals the number of channels flowing out (64, as shown in Figure 3). This, like ResNet, aids in improving gradient flow during the rearward pass. Since it passes between the bottleneck layers which have a limited number of channels, the authors of MobileNetv2 refer to this as an inverted residual. A standard ResNet residual link, on the other hand, connects layers with multiple channels. The activation function, as previously mentioned, is ReLU6. Behind the bottleneck layer, however, there is no activation. As a result, the paper’s title refers to linear bottlenecks. Since this layer creates low-dimensional data, the scientists discovered that adding a non-linearity removes important information. All of these basic elements make up the MobileNetv2 architecture. A standard 1 × 1 convolution, a global average pooling layer, and a classification layer are then applied as illustrated in Figure 3.

Table 1 showcases the Top1 and Top5 accuracies of various lightweight CNN architectures.

**Table 1:** Comparison of various lightweight architectures.

Architecture	Year	Parameters	Top1	Top5
ShuffleNetv2 [22]	2018	2.3 M	69.4	88.9
MobileNetv2 [25]	2018	3.47 M	71.8	91.0
ESPNetv2 [23]	2019	3.49 M	72.06	90.39
DiCENet [24]	2020	2.65 M	69.05	88.8

In Section 3, DBGC—Dimension-based Generic Convolution block, is proposed in this paper which requires complete understanding of various concepts such as separable convolution and various convolution kernels outlined in Section 2 which is subdivided into two parts: the first part explains the separable convolution method and its types; the second part explains the various convolutional kernels. Section 4 includes results and analysis for the proposed DBGC block.

## 2. Materials and Methods

This section explains required terminologies and their importance for the proposed DBGC block. This section is divided into separable convolution and depth-wise separable convolution.

### 2.1. Introduction to Separable Convolution

The concept of separable convolution was first introduced in the Xception model in 2016 [28]. Researchers provided multiple approaches to make inferential computation more efficient on smart phones and IoT Devices such as network pruning, parameter compression, and so on, as deep learning (DL) is increasingly pushing toward edge computing. Quantization, as one of the primary ways, may effectively offload GPU, allowing DL to run on a fixed-point pipeline. The popular lightweight MobileNetv1 significantly decreases parameter size and computation lag using the concept of separable convolution [29].

Separable convolutions are of the following two types: (1) spatial separable convolution, and (2) depth-wise separable convolution. 

#### 2.1.1. Spatial Separable Convolution

**Figure 4:**
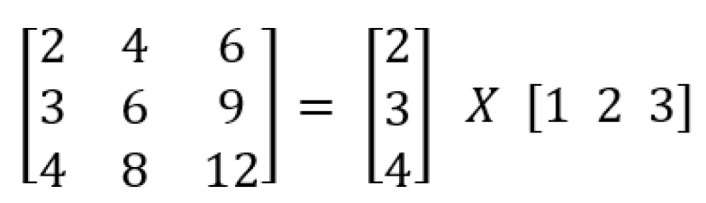
Splitting a 3 × 3 kernel spatially.

Spatial separable convolution is the easier of the two to understand conceptually, and it clearly demonstrates the concept of splitting one convolution into two [28]. Unfortunately, spatial separable convolutions have a number of drawbacks, thus they are not widely employed in deep learning. The “spatial separable convolution” name originates from the fact that it primarily works with the width and height of an image and kernel [30] (the number of channels in each image is the other dimension, the “depth” dimension). Spatial separable convolution separates a kernel into two smaller kernels. The most typical scenario is to split a 3 × 3 kernel into 3 × 1 and 1 × 3 kernels as shown in Figure 4.

**Figure 5:**
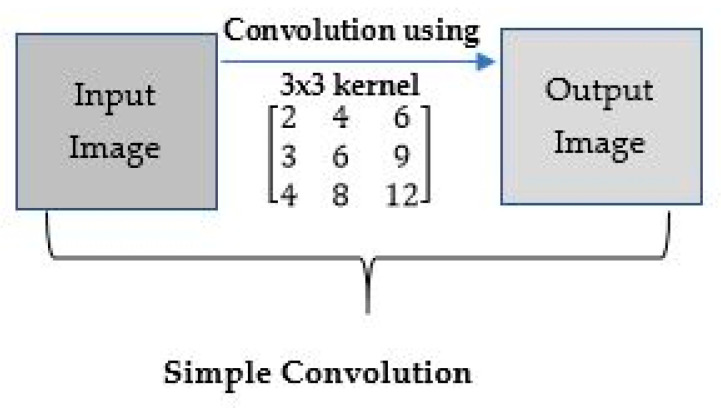

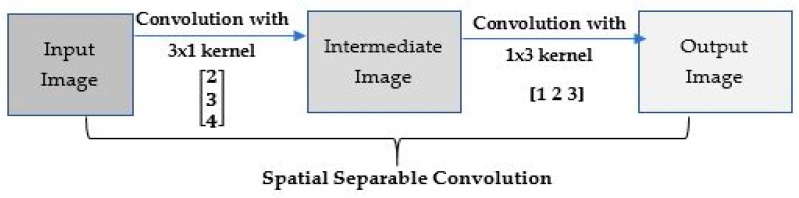
Simple and Spatial Separable Convolution.

To accomplish the same result, instead of doing one convolution with nine multiplications, perform two convolutions with three multiplications each which are six in total. Computational complexity decreases as the number of multiplications decreases, allowing the network to operate more quickly as shown in Figure 5.

The main drawback of a spatial separable kernel is that it may not split all the kernels in two. This becomes tedious during training as it only uses a tiny portion of the whole network.

#### 2.1.2. Depth-Wise Separable Convolution

This convolution works with kernels that cannot be “factored” into two smaller kernels, which is the main reason behind the popularity of depth-wise separable convolution. It can be implemented in keras with keras.layers.SeparableConv2D or tf.layers.separable_conv2d. 

The depth-wise separable convolution obtains its name from the fact that it works with both spatial and depth dimensions (the number of channels) [31] Three channels are possible in an input image: R, G, and B. A picture may contain numerous channels after a few convolutions [32]. Each channel may be thought of as a different interpretation of the image; the “red” channel, for example, interprets the “redness” of each pixel; the “blue” channel interprets the “blueness” of each pixel; and the “green” channel interprets the “greenness” of each pixel. A picture with 64 channels can be interpreted in 64 distinct ways. A depth-wise separable convolution, such as a spatial separable convolution, seperates a kernel into two independent kernels that perform two convolutions: depth-wise and point-wise.

##### Depth-Wise Convolution

**Figure 6:**
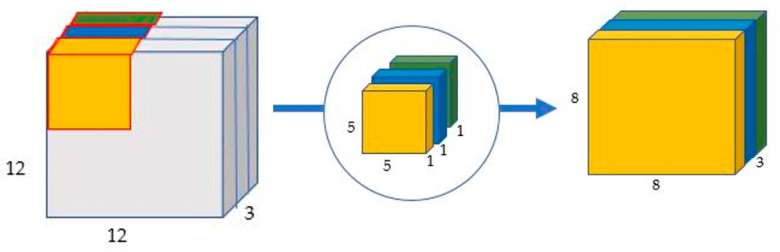
Depth-wise convolution uses three kernels to produce an 8 × 8 × 1 image from a 12 × 12 × 1 image.

In depth-wise convolution the input image is provided with convolution kernels without changing its depth [33]. This task is done using three kernels. Figure 6 provides an example. Here, a 12 × 12 × 3 image is provided as an input image and three kernels are used each with shape 5 × 5 × 1. Each 5 × 5 × 1 kernel iterates one channel of the provided image, provides the scalar products of every 25 (5 × 5) pixel group, and provides the output with an 8 × 8 × 1 image as shown in Figure 6.

##### Point-Wise Convolution

The original convolution takes a 12 × 12 × 3 image and generates an image of dimensions 8 × 8 × 256. The depth-wise convolution converts the 12 × 12 × 3 image into an 8 × 8 × 3 image. The number of channels in each image must then be increased.

**Figure 7:**
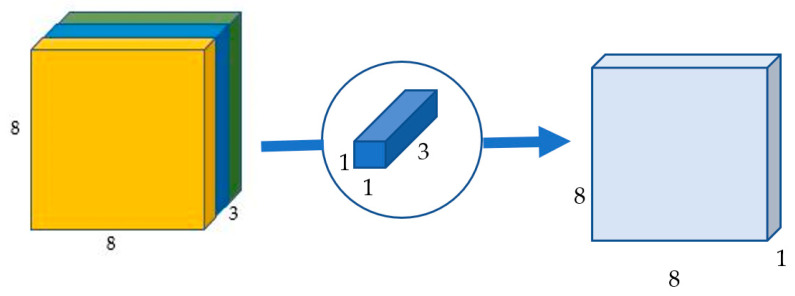
Point-wise convolution transforms an image of three channels into an image of one channel.

The point-wise convolution name originates from the fact that it employs a 1 × 1 kernel, which iterates across every single point. This kernel has a depth equal to the number of channels in the input picture; for the provided example it will be three [34]. In order to generate an 8 × 8 × 1 image, a 1 × 1 × 3 kernel iterates across the 8 × 8 × 3 image as shown in Figure 7.

To check the number of multiplications the computer has to perform in the normal convolution, which includes 256 kernels (each of size 5 × 5 × 3), and moves 8 × 8 times. Thus, it yields 256 × (5 × 5 × 3) × 8 × 8 = 1,228,800 multiplications. Meanwhile in separable convolution there are three 5 × 5 × 1 kernels that move 8 × 8 times, thus in depth-wise convolution there are 3 × 5 × 5 × 8 × 8 = 4800 multiplications. We have 256 × 1 × 1 × 3 kernels that move 8 × 8 times in the point-wise convolution. Thus, it will be 256 × 1 × 1 × 1 × 3 × 8 × 8 = 49,152 multiplications. Therefore, finally by adding 4800 + 49,152 it produces 53,952 multiplications altogether. 1,228,800 is a lot more than 53,952. The network can handle more data in less time by performing fewer calculations.

The fundamental difference between standard convolution and depth-wise separable convolution is that in standard convolution the picture is changed 256 times and each change needs a total of 4800 multiplications (5 × 5 × 3 × 8 × 8). Meanwhile, in separable convolution an image is truly altered only once and after that, the altered image is lengthened to 256 channels. Thus, processing resources are saved. The only demerit of separable convolution is that it minimizes the number of parameters in a convolution; if a network is already tiny, it may wind up with too few parameters, causing it to fail to learn effectively during training. However, when utilized correctly it tends to increase efficiency without sacrificing efficacy, making it a popular choice.

### 2.2. Introduction to Convolution Kernels

**Figure 8:**
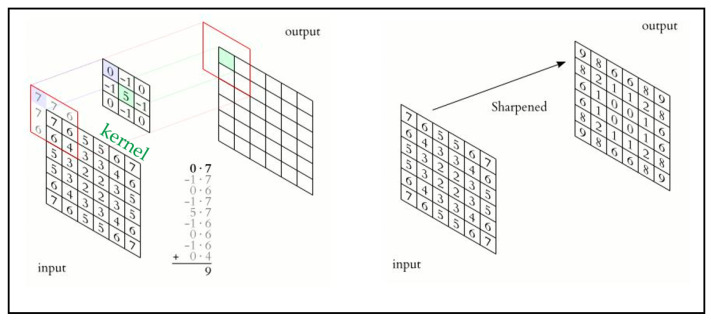
Example of convolution using a kernel.

Convolution means using a ‘kernel’ to extract certain ‘features’ from an input image. A kernel is a matrix which is slid across the image and multiplied with the input such that the output is enhanced in a certain desirable manner as shown in Figure 8. The following example demonstrates the use of a kernel for making the input image sharpened in the sense of having the output image represented in the most desirable manner.

A kernel is a matrix of weights multiplied by the input to extract important features. The name of the convolution comes from the dimensions of the kernel matrix. The kernel matrix in 2D convolutions, for example, is a 2D matrix. A filter, on the other hand, is a concatenation of numerous kernels, each of which is allocated to a certain input channel. Filters usually have one more dimension than kernels. Filters in 2D convolutions, for example, are 3D matrices. The filter dimensions for a CNN layer with kernel dimensions of h*w and input channels of k are k*h*ws. On the basis of various kernels there are three types of convolutions: (1) 1D convolution, (2) 2D convolution, and (3) 3D convolution.

#### 2.2.1. 1D Convolution

**Figure 9:**
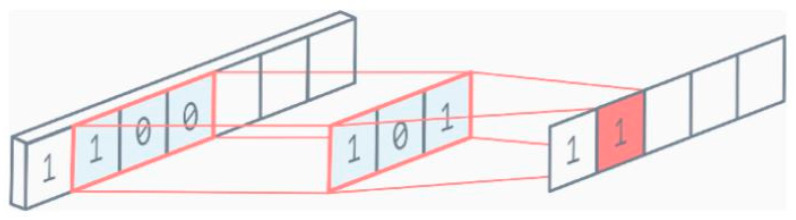
1D convolution.

For time series data processing, 1D convolutions are extensively utilized (since the input in such cases is 1D). The 1D data input, as previously stated, might include many channels [35]. Since the filter can only travel in one direction, the output is 1D. A single channel 1D convolution example is shown in Figure 9.

#### 2.2.2. 2D Convolution

**Figure 10:**
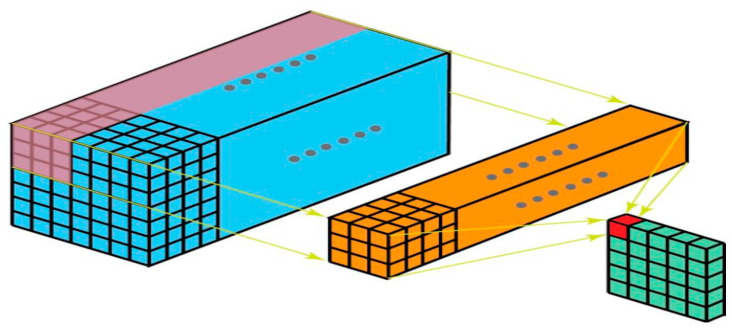
2D convolution.

The kernel dimensions in Figure 10 below are 3 × 3 and there are numerous such kernels in the filter (marked yellow). This is due to the fact that the input has numerous channels (indicated in blue) and there is one kernel for each channel. Clearly, the filter may travel in two directions in this case and the final output is two-dimensional. The most frequent convolutions are 2D convolutions, which are widely utilized in computer vision.

#### 2.2.3. 3D Convolution

**Figure 11:**
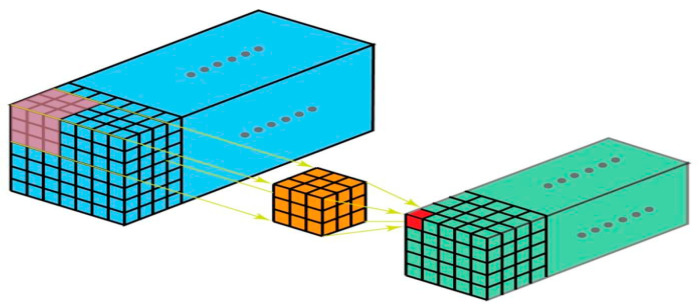
3D convolution.

Since a 3D filter (which is a 4D matrix) is difficult to visualize, we will only cover single channel 3D convolution here. As can be seen in Figure 11 below, a kernel in a 3D convolution can travel in three directions, resulting in 3D output [36]. The majority of research done on customizing and altering CNN layers has been limited to 2D convolutions.

## 3. DBGC—Dimension-Based Generic Convolution Unit

Standard convolutions simultaneously encode spatial and channel-wise information, but they are computationally intensive. Separable (or depth-wise separable) convolutions are used to increase the efficiency of ordinary convolutions by encoding spatial and channel-wise information separately using depth-wise and point-wise convolutions, respectively [37]. Although separable convolutions are efficient during factorization, they place a large computational burden on point-wise convolutions and make them a computational bottleneck [24].

To encode spatial and dimension-wise information efficiently, the DBGC unit uses a dimension selector to further reduce any bottleneck issues and also reduce computational load by introducing the dimension selector module discussed in Section 3.2. It completes this task using two stages as shown in Figure 8. One stage is a convolution based on dimension as explained in Section 3.1, and the second stage is a dimension-wise blend as discussed in Section 3.3. Convolution based on dimension enables the DBGC unit to use a dimension-wise blend instead of using point-wise convolutions that create computational bottlenecks.

### 3.1. Convolution Based on Dimension (ConvDim)

**Figure 12:**
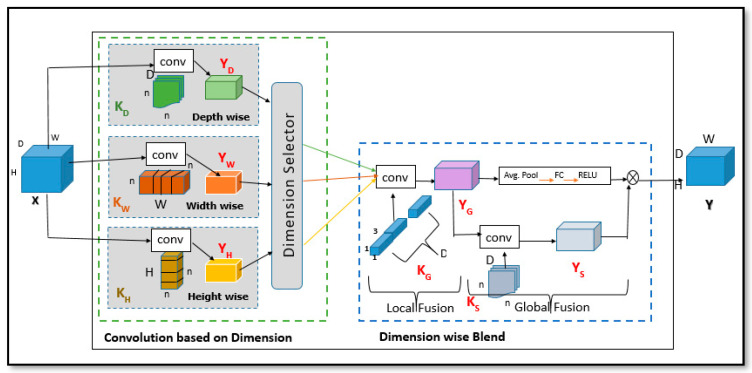
DBGC architecture.

ConvDim block encodes information independently that is height-wise, depth-wise and width-wise. In order to accomplish this, ConvDim extends depth-wise separable convolutions to all dimensions of the input tensor I ∈ R ^H × D × W^, where H, D and W correspond to height, depth and width of I, respectively. As shown in Figure 12, ConvDim has three kernels, one for each dimension. They apply various dimension-wise kernels, such as H height-wise convolutional kernel K_H_ ∈ R ^1 × n × n^ along height; D depth-wise convolutional kernel K_D_ ∈ R ^n × 1 × n^ along depth; and W width-wise convolutional kernel K_W_ ∈ R ^n × n × 1^ along width. Those kernels produce outputs denoted as Y_H_, Y_D_ and Y_W_ ∈ R ^H × D × W^ which encode information provided in the input tensor. The outputs of these independent branches are concatenated in the dimension selector block, such that the first spatial plane of YD, YW, and YH are put together and so on, to produce the output YDim.

### 3.2. Dimension Selector (Ds)

**Figure 13:**
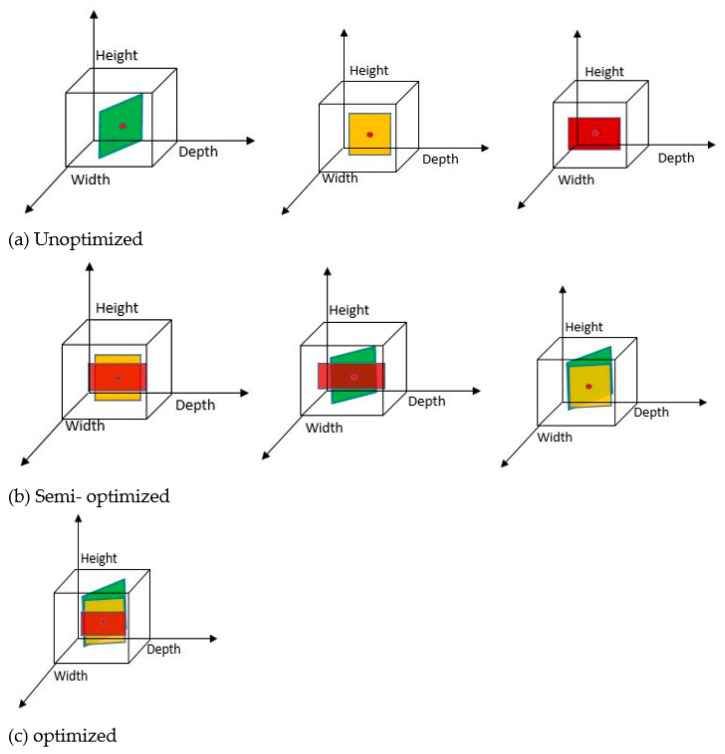
Implementation of Convolution based on Dimension (ConvDim): in (**a**), each kernel is applied to a pixel (represented by a small dot) independently; in (**b**), any two kernels are (height-width, height-depth, and width-depth) applied to a pixel simultaneously by allowing information to be combined using tensors; finally in (**c**), all kernels are applied to a pixel simultaneously, allowing information to be aggregated from the tensor efficiently. Convolutional kernels are highlighted in color (depth-wise, width-wise, and height-wise).

Dimension selector is the block where dimensions can be selected. If an application is such that only height, or only width, or only depth dimensions are enough for training then one can set the parameters from this block, Ds ∈ {K_D_ ∪ K_W_ ∪ K_H_}. It is also possible to select any two combinations of kernels such that Ds ∈ {K_D_, K_W_ ∪ K_H_, K_W_ ∪ K_D_, K_H_}, where Ds is Dimension selector and K_D_, K_H_, K_W_ are dimension-based kernels (depth, width, and height, respectively), Ds ∈ {K_D_, K_W_, K_H_}. So, in Dimension selector there are total of seven possibilities (only height; only width; only depth; height and width; width and depth; height and depth; and height and width and depth). Based on the selection of kernels, the appropriate dimensions will be provided to YDim. Various dimensions can be selected as shown in Figure 13.

### 3.3. Dimension-Wise Blends (DimBlend)

Local information from distinct dimensions of the input tensor is encoded by dimension-wise convolutions, but global information is not captured. Local means each selected dimension-wise information and global means information collected from each dimension. A point-wise convolution is a common method for combining information globally in CNNs [3,7]. In order to combine dimension-wise representations of Y_Dim_ ∈ R^3DXHXW^ and create an output Y ∈ R ***^DXHXW^***, a point-wise convolutional layer applies point-wise kernels Kp ∈ R ^3DX1X1^ and executes 3D^2^HW operations. This takes a lot of time to compute. However, the dimension-wise blend module allows one to mix representations of Y_Dim_ effectively, given DimConv’s capacity to encode spatial and channel-wise information (though separately). DimBlend factorizes the point-wise convolution in two phases, as shown in Figure 12: (1) local fusion and (2) global fusion [26]. 

In local fusion Y_Dim_ concatenates the output from the dimension selector module. It concatenates spatial planes of each dimension. DimBlend uses a group point-wise convolution layer in order to combine dimension-wise information received from Y_Dim_. As shown in Figure 12, Kg operates independently on each dimension group D. This process is denoted as local fusion.

As indicated in Figure 8, DimBlend learns channel-wise and spatial representation independently and then propagates channel-wise encodings to spatial encoding by applying element-wise multiplication in order to effectively encode global information in Yg. As outlined in [38], spatial dimensions of Yg are squeezed and encoded into channel-wise presentation by utilizing two FC Layers. The First FC Layer reduces the dimension from D to D/4 and the second FC Layer expands the dimension from D/4 to D. The ReLU function is used in between these two FC Layers to make them learn about non-linear representations. This process is known as global fusion. By combining local and global fusion in DimBlend, final output Y is produced.

## 4. Results and Analysis

This section provides detail about implementation of the proposed DBGC block and further provides results analysis for it.

### 4.1. Implementation of DBGC

**Figure 14:**
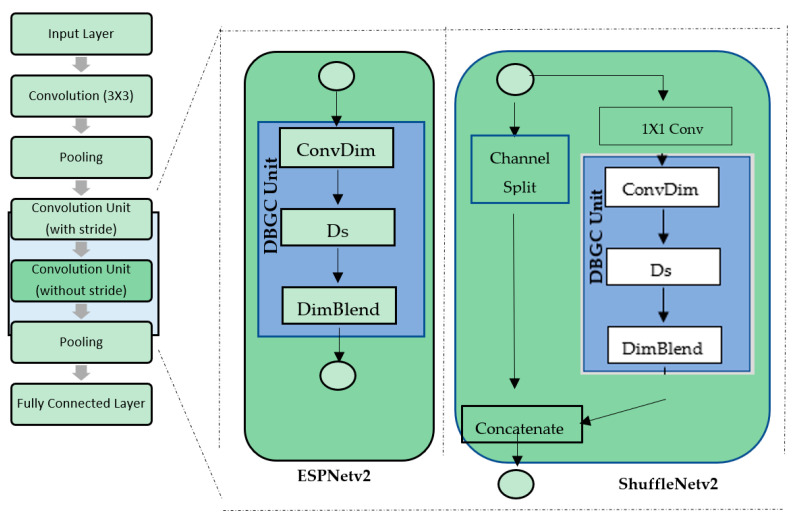
**The** DBGC unit in different architecture (ESPNetv2 and ShuffleNetv2) designs.

To implement the DBGC unit in CNN, conventional CNN layers are used. Figure 14 showcases the overall architecture.

#### 4.1.1. Experimental Setup

By applying the DBGC Block on ESPNetv2 [23] and ShuffleNetv2 [22] architecture, we evaluated the generic nature of the DBGC unit on the PASCAL VOC dataset explained in Section 4.2. We integrated the DBGC unit into different architectures as shown in Figure 10 and studied the impact on FLOPs and accuracy. Section 5 explains the analysis of using the DBGC unit with various architectures.

#### 4.1.2. Dataset Details

To demonstrate the performance of the DBGC unit on various models, a common dataset was taken in order to have directly comparable results for the same dataset using different architectures. We used the PASCAL VOC (PASCAL Visual Object Classes Challenge) dataset for implementation purposes. Aeroplane, bicycle, boat, bus, car, motorcycle, train, bottle, chair, dining table, potted plant, sofa, TV/monitor, bird, cat, cow, dog, horse, sheep, and person are among the 20 object categories in the PASCAL Visual Object Classes (VOC) 2012 dataset [39]. There are pixel-level segmentation annotations, bounding box annotations, and object class annotations for each image in this dataset. Object detection, semantic segmentation, and classification applications have all utilized this dataset as a standard. The PASCAL VOC dataset is divided into three parts: training pictures, validation images, and a private testing set.

### 4.2. Results Analysis

This section describes the results of the two architectures (ESPNetv2 and SHUFFLENetv2) used with the DBGC unit. The primary goal was to observe effects on the accuracy of object detection and semantic segmentation when reducing FLOPs. The ESPNetv2 model was used to implement object detection while semantic segmentation was achieved with ShuffleNetv2. It is important to understand two main terminologies, FLOPs and FLOPS.

FLOPs refers to what a model will have to execute to determine inference times in order to compute the total amount of calculations. This is when the term FLOP, or Floating Point Operation, emerges [40]. This might be any operation that uses a floating-point value, such as addition, subtraction, division, or multiplication. FLOPs determine the model’s complexity.

FLOPS, with a capital S, is Floating Point Operations per Second (FLOPS), and is a unit of measurement. It is a metric that indicates how good the hardware is. The faster a model can perform operations per second, the faster it can infer. Equations used to calculate FLOPs in the proposed model are illustrated in Table 2.

This section is divided into three categories: (a) unoptimized kernel dimensions, (b) semi-optimized kernel dimensions, and (c) optimized kernel dimensions. Unoptimized kernel dimensions include only height, only width, and only depth-based kernel selection during the Dimension selector module. Semi-optimized kernel dimensions include height-width, width-depth, and depth-height combinations during the dimension selection phase. Optimized kernel dimensions provide all three dimensions during the dimension selection phase.

#### 4.2.1. Unoptimized Kernel Dimensions

In unoptimized kernel dimensions, only one kernel dimension for the output channel is used. It is very clear that this will definitely reduce the accuracy of object detection. ESPNetv2 architecture was used for object detection purposes and ShuffleNetv2 was used for semantic segmentation. The PASCAL VOC dataset was used in order to first implement ESPNetv2 and ShuffleNetv2 as outlined in [24]. Then the same models were used with proposed DBGC-Kw; that is, the DBGC block was implemented and in the dimension selector module the width parameter was selected as described in the DBGC architecture from Section 3. The main aim was to observe differences in FLOPs and to check its Top1 and Top5 accuracies. Table 3, Table 4 and Table 5 demonstrate the results obtained using only width-based, height-based, and depth-based kernels, respectively.

**Figure 15:**
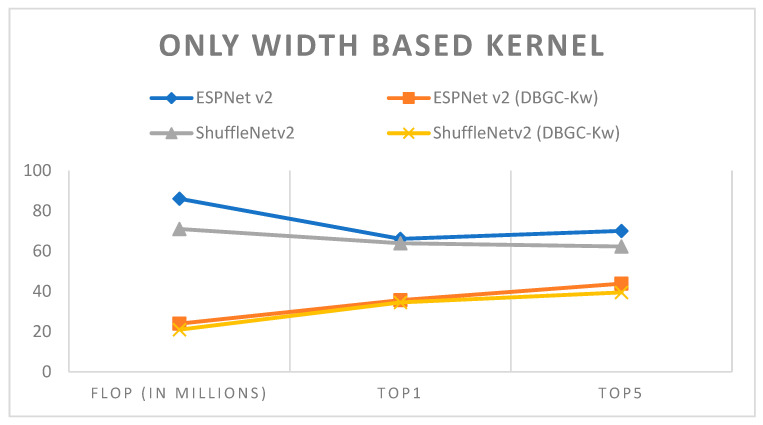
Only width-based kernel.

**Figure 16:**
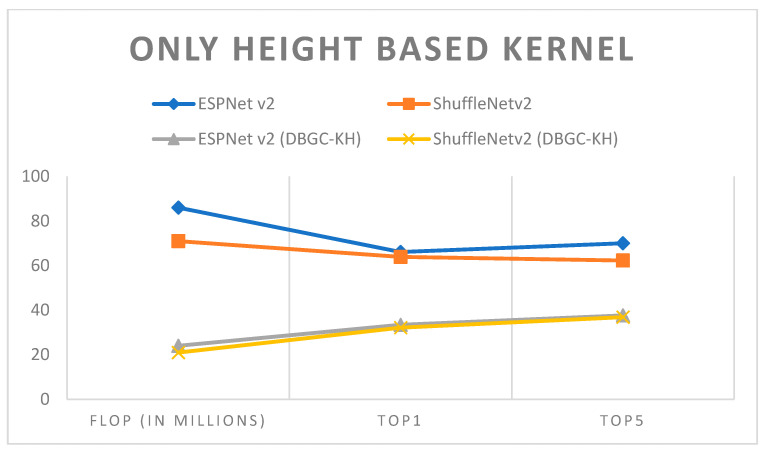
Only height-based kernel.

**Figure 17:**
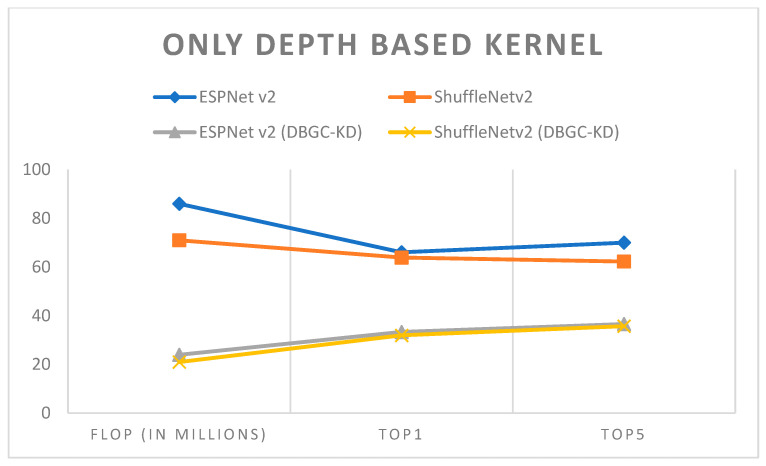
Only depth-based kernel.

**Figure 18:**
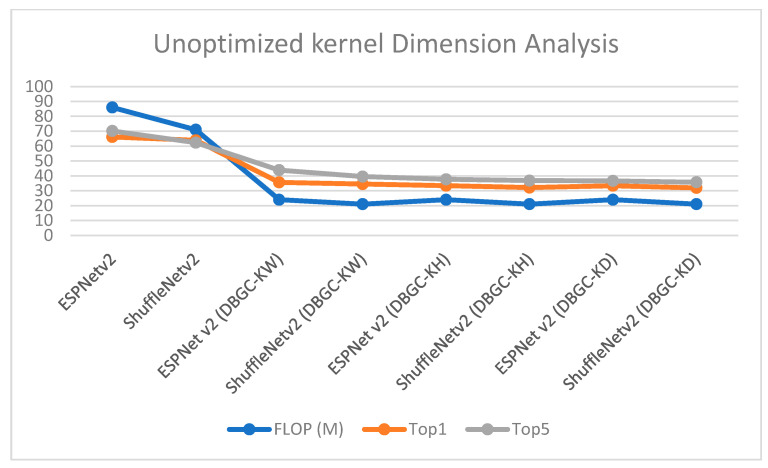
Analysis of unoptimized kernel dimensions.

It can be observed from the results that unoptimized kernel dimensions reduce FLOPs to around one third of the FLOPs required to implement original ESPNetv2 or ShuffleNetv2 architecture. At the same time it can also be noticed that if we use only a single dimension, accuracy is affected very negatively. Accuracy is reduced by around 30%. It can be easily recognized from Figure 15, Figure 16, Figure 17 and Figure 18.

In order to analyze the results, Figure 18 was created. It denotes that by reducing dimensions FLOP decreases drastically but at the same time it also negatively effects accuracy. Reducing dimensions reduces accuracy by around 50%, which is the reason that this method is not suitable for real-world applications. 

#### 4.2.2. Semi-Optimized Kernel Dimensions

In semi-optimized kernel dimensions, we used a combination of two kernel dimensions for the output channel. It is very clear that this definitely reduces the number of FLOPs without affecting the accuracy significantly. ESPNetv2 architecture was used for object detection purposes and ShuffleNetv2 was used for semantic segmentation. The PASCAL VOC dataset was used in order to first implement ESPNetv2 and ShuffleNetv2 as outlined in [24]. Then the same models were used with proposed DBGC-Kwh, DBGC-Kdh and DBGC-Kwd; that is, the DBGC block was implemented in the dimension selector modules for width and height, or width and depth, or height and width. Parameters were selected as described in the DBGC architecture from Section 3. The main aim was to look for differences in FLOPs and to check Top1 and Top5 accuracies. Table 6, Table 7 and Table 8 demonstrate the results obtained using depth + width-based, depth + height-based and height + width-based kernels, respectively.

**Figure 19:**
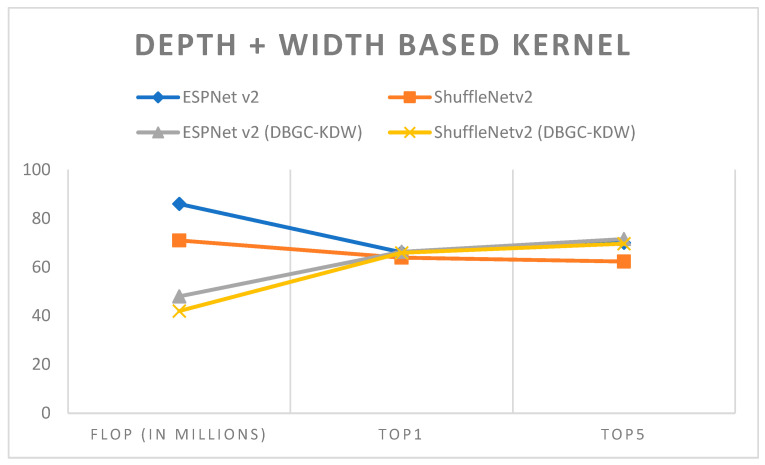
Depth + width-based kernel.

**Figure 20:**
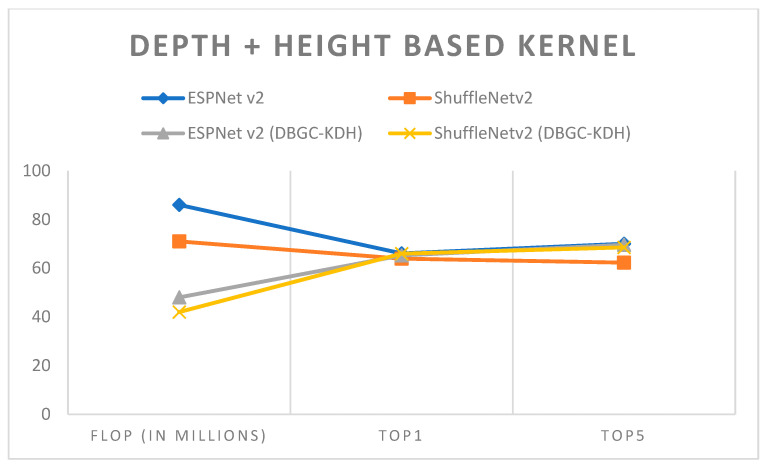
Depth + height-based kernel.

**Figure 21:**
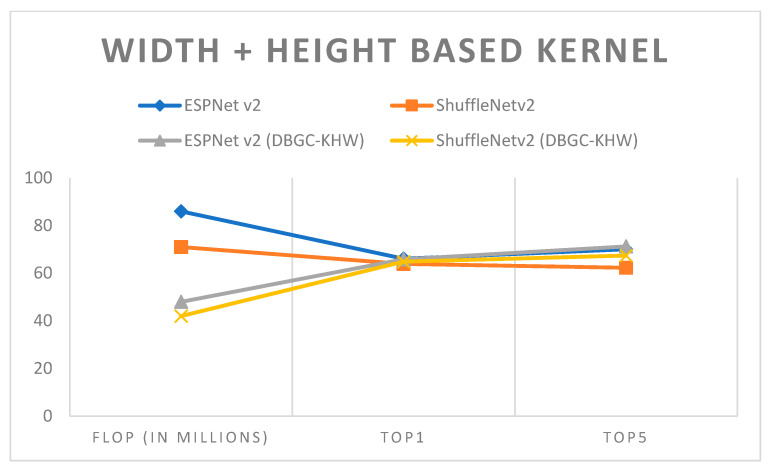
Height + width-based kernel.

It can be observed from the results that semi-optimized kernel dimensions reduce FLOPs to around half of the FLOPs required to implement the original ESPNetv2 or ShuffleNetv2 architecture. At the same time it can also be noticed that if we use any two dimensions, DBGC provides good accuracy. The accuracy is almost the same with half the FLOPs which is a really good indication that decent speeds can be achieved with almost the same accuracy in object detection, and even 1 or 2% higher accuracy in semantic segmentation. This can be easily recognized in examining Figure 19, Figure 20 and Figure 21.

**Figure 22:**
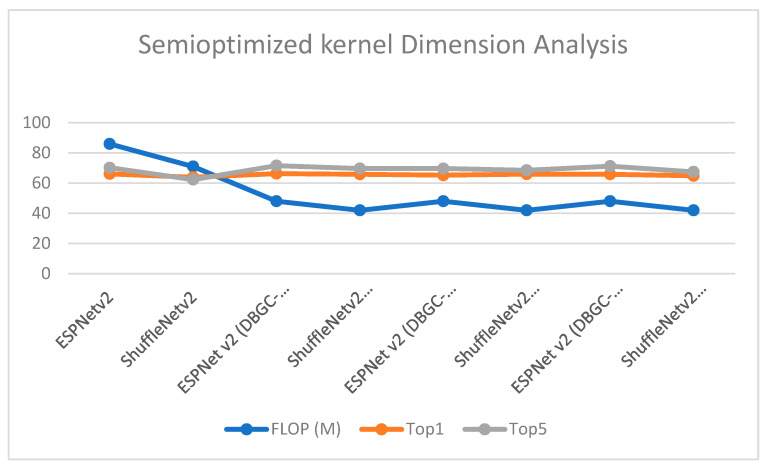
Semi-optimized kernel dimension.

In order to analyze these results, Figure 22 was created. It denotes that by using two dimensions, FLOP decreases but at the same time accuracy is achieved nearly equal to that of the original architecture. Thus, it is a positive sign that we were able to reduce computations as FLOP was reduced without compromising accuracy. Such semi-optimized kernel dimension mechanisms can be used in mobile networks or even for sensors and in IoT where there are requirements of low power, low computations, and low storage.

#### 4.2.3. Optimized Kernel Dimension

**Figure 23:**
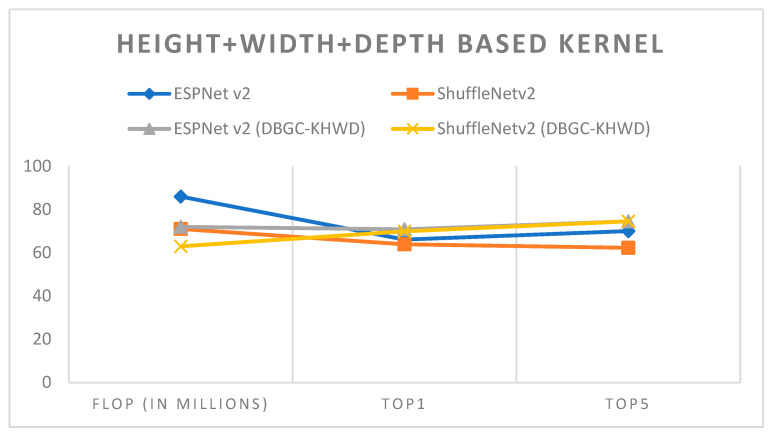
Height + width + depth-based kernel.

In optimized kernel dimensions, we used a combination of all three kernel dimensions for the output channel. It is very clear that this definitely provided very good accuracy but with higher numbers of FLOPs also. DBGC-Khwd reduced the number of FLOPs with better accuracy. ESPNetv2 architecture was used for object detection purposes and ShuffleNetv2 was used for semantic segmentation. The PASCAL VOC dataset was used in order to first implement ESPNetv2 and ShuffleNetv2 as outlined in [24]. Then the same models were used with proposed DBGC-Khwd; that is, the DBGC block was implemented and in the dimension selector module width, height, and depth parameters were selected as described in the DBGC architecture from Section 3. The main aim was to look for differences in FLOPs and to check Top1 and Top5 accuracies. Table 9 and Figure 23 demonstrate the results obtained using depth + width + height-based kernels parallel.

It can be recognized from Figure 20 that by selecting all three dimensions a 4 to 5% increase in accuracy was achieved while reducing FLOPs. Optimized kernels yield the best results among all three categories of kernel dimensions.

**Figure 24:**
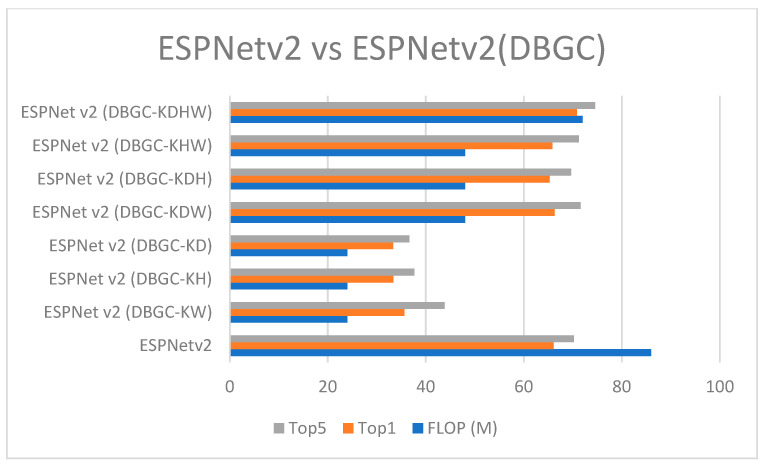
ESPNetv2 versus ESPNetv2 (DBGC).

**Figure 25:**
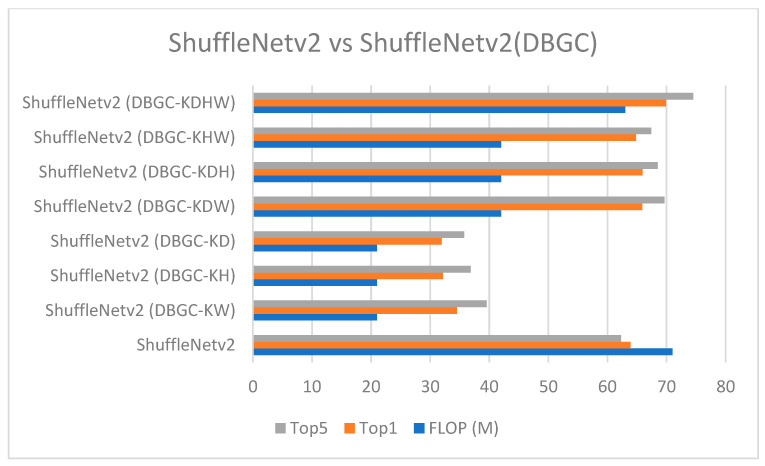
ShuffleNetv2 versus ShuffleNetv2 (DBGC).

Figure 24 and Figure 25 demonstrate the comparison of all unoptimized, semi-optimized, and optimized kernel dimensions in ESPNetv2 and ShuffleNetv2, respectively. 

**Figure 26:**
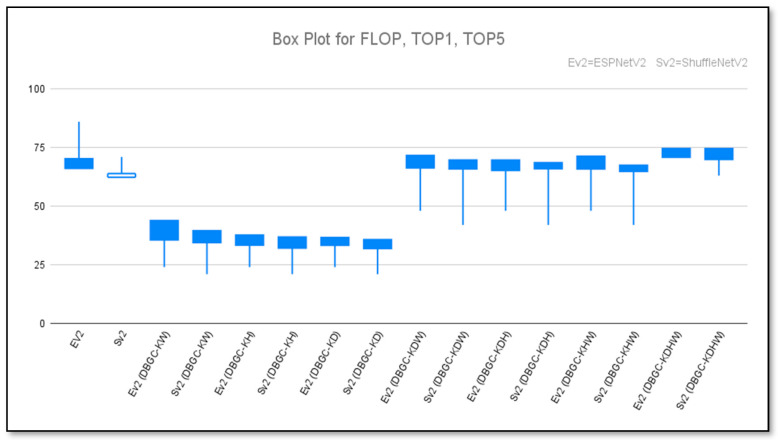
Box plot to show unoptimized, semi-optimized, and optimized kernel performances for ESPNetv2 versus ESPNetV2 (DBGC) and ShuffleNetv2 versus ShuffleNetV2 (DBGC).

Figure 26 shows a box plot of all the methods used for the PASCAL VOC dataset. “Ev2” is used as the short form of ESPNetv2 and “Sv2” is used for ShuffleNetv2.

**Figure 27:**
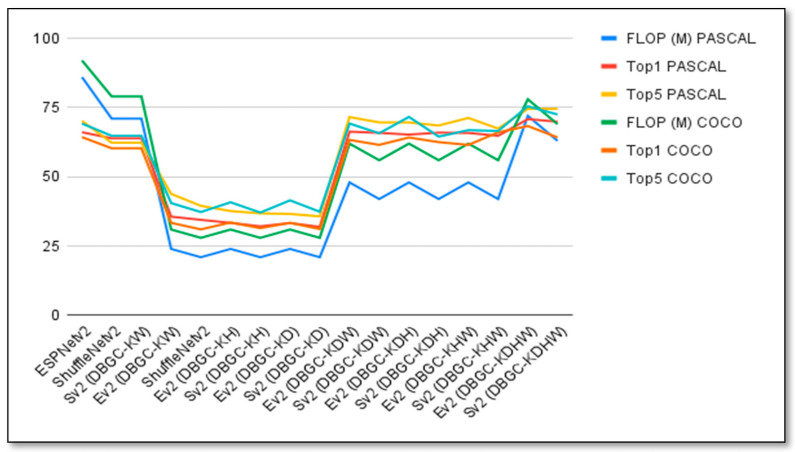
Graph to visualize the performances of unoptimized, semi-optimized, and optimized kernels for ESPNetv2 versus ESPNetV2 (DBGC), and ShuffleNetv2 versus ShuffleNetV2 (DBGC) using the PASCAL and COCO datasets.

To cross validate the performances of the proposed DBGC it was also used with the MS COCO dataset [41]. Table 10 shown below displays the performance of unoptimized, semi-optimized, and optimized kernel for ESPNetv2 versus ESPNetV2 (DBGC) and ShuffleNetv2 versus ShuffleNetV2 (DBGC) using the MS COCO dataset. The chart for the same is displayed in Figure 27.

## 5. Conclusions

The proposed DBGC unit is generic and can be used with any CNN-based network model. DBGC was used with ESPNetv2 and ShuffleNetv2 architectures. The results were evaluated on the basis of FLOPs and Top1 and Top5 accuracies. All the practical implementation was performed on the PASCAL VOC dataset. It can be concluded that the unoptimized kernel-based DBGC provides around one third less FLOPs which increases speed drastically; however, at the same time accuracy is reduced drastically. Semi-optimized dimension-based kernels provide around half the FLOPS with the same or greater accuracy in ShuffleNetV2 with DBGC. Optimized dimension-based kernels provide the highest accuracy with FLOPs reduced by around 5 M.

## 6. Future Work

In future studies the same architecture could be tested using various datasets. Arithmetically it could be determined if unoptimized dimension-based kernels applied on single-dimension data could provide better accuracy. In DBGC, dimensions are selected manually but future dimension selector modules could be optimized to select dimensions based on the particular datasets provided.

## Figures and Tables

**Table 2:** Equations to calculate FLOPs of each CNN layer.

Sr. No	Layer	The Equation to Calculate FLOPs
1	Convolution Layer	2 × No. of Kernel × Kernel’s Shape × Output Shape × Repeat Count (if available)
2	Pooling Layer (Without stride)	Height × Width × Depth of an input Image
3	Pooling Layer (With stride)	(Height/Stride) × Depth × (Width/Stride) of an input Image
4	Fully Connected Layer (FC Layer)	2 × Input Size × Output Size

Output shape of Convolution Layer can be determined using following equation, Output Shape = (Input Shape − Kernel Shape) + 1.

**Table 3:** Only width-based kernel.

Model	Dataset	Image Size	FLOP (In Millions)	Top1	Top5
**ESPNet v2**	PASCAL	**224 × 224**	**86**	**66.1**	**70.02**
**ShuffleNetv2**	PASCAL	224 × 224	**71**	**63.9**	**62.30**
**ESPNetv2(DBGC-Kw)**	PASCAL	224 × 224	**24**	**35.64**	**43.86**
**ShuffleNetv2 (DBGC-Kw)**	PASCAL	224 × 224	**21**	**34.5**	**39.54**

**Table 4:** Only height-based kernel.

Model	Dataset	Image Size	FLOP (In Millions)	Top1	Top5
**ESPNet v2**	PASCAL	224 × 224	**86**	**66.1**	**70.02**
**ShuffleNetv2**	PASCAL	224 × 224	**71**	**63.9**	**62.30**
**ESPNetv2(DBGC-KH)**	PASCAL	224 × 224	**24**	**33.4**	**37.66**
**ShuffleNetv2 (DBGC-KH)**	PASCAL	224 × 224	**21**	**32.15**	**36.84**

**Table 5:** Only depth-based kernel.

Model	Dataset	Image Size	FLOP (In Millions)	Top1	Top5
**ESPNet v2**	PASCAL	224 × 224	**86**	**66.1**	**70.02**
**ShuffleNetv2**	PASCAL	224 × 224	**71**	**63.9**	**62.30**
**ESPNetv2(DBGC-KD)**	PASCAL	224 × 224	**24**	**33.34**	**36.62**
**ShuffleNetv2 (DBGC-KD)**	PASCAL	224 × 224	**21**	**31.95**	**35.74**

**Table 6:** Depth + width-based kernel.

Model	Dataset	Image Size	FLOP (In Millions)	Top1	Top5
**ESPNet v2**	PASCAL	**224 × 224**	**86**	**66.1**	**70.02**
**ShuffleNetv2**	PASCAL	224 × 224	**71**	**63.9**	**62.30**
**ESPNetv2(DBGC-KDW)**	PASCAL	224 × 224	**48**	**66.31**	**71.58**
**ShuffleNetv2 (DBGC-KDW)**	PASCAL	224 × 224	**42**	**65.88**	**69.65**

**Table 7:** Depth + height-based kernel.

Model	Dataset	Image Size	FLOP (In Millions)	Top1	Top5
**ESPNet v2**	PASCAL	**224 × 224**	**86**	**66.1**	**70.02**
**ShuffleNetv2**	PASCAL	224 × 224	**71**	**63.9**	**62.30**
**ESPNetv2(DBGC-KDH)**	PASCAL	224 × 224	**48**	**65.25**	**69.63**
**ShuffleNetv2 (DBGC-KDH)**	PASCAL	224 × 224	**42**	**65.95**	**68.53**

**Table 8:** Height + width-based kernel.

Model	Dataset	Image Size	FLOP (In Millions)	Top1	Top5
**ESPNet v2**	PASCAL	**224 × 224**	**86**	**66.1**	**70.02**
**ShuffleNetv2**	PASCAL	224 × 224	**71**	**63.9**	**62.30**
**ESPNetv2(DBGC-KHW)**	PASCAL	224 × 224	**48**	**65.85**	**71.25**
**ShuffleNetv2(DBGCKHW)**	PASCAL	224 × 224	**42**	**64.82**	**67.43**

**Table 9:** Height + width + depth-based kernel.

Model	Dataset	Image Size	FLOP (In Millions)	Top1	Top5
**ESPNet v2**	PASCAL	**224 × 224**	**86**	**66.1**	**70.02**
**ShuffleNetv2**	PASCAL	224 × 224	**71**	**63.9**	**62.30**
**ESPNetv2(DBGC-KHWD)**	PASCAL	224 × 224	**72**	**70.83**	**74.56**
**ShuffleNetv2 (DBGC-KHWD)**	PASCAL	224 × 224	**63**	**69.92**	**74.53**

**Table 10:** Unoptimized, semi-optimized, and optimized kernel performances for ESPNetv2 versus ESPNetV2 (DBGC) and ShuffleNetv2 versus ShuffleNetV2 (DBGC) for the PASCAL and COCO datasets.

	Dataset	ESPNetv2 (Ev2)	ShufleNetv2 (Sv2)	Ev2(DBGC-K_W_)	Sv2 (DBGC-K_W_)	Ev2(DBGC-KH)	Sv2(DBGC-KH)	Ev2(DBGC-KD)	Sv2(DBGC-KD)	Ev2(DBGC-KDW)	Sv2(DBGC-KDW)	Ev2(DBGC-KDH)	Sv2(DBGC-KDH)	Ev2(DBGC-KHW)	Sv2(DBGC-KHW)	Ev2(DBGC-KDHW)	Sv2(DBGC-KDHW)
**FLOP (M)**	PASCAL	86	71	24	21	24	21	24	21	48	42	48	42	48	42	72	63
**Top1**		66.1	**63.9**	35.64	34.5	33.4	32.15	33.34	31.95	66.31	65.88	65.25	65.95	65.85	64.82	70.83	69.92
**Top5**		70.2	**62.3**	43.86	39.54	37.66	36.84	36.62	35.74	71.58	69.65	69.63	68.53	71.25	67.43	74.56	74.53
**FLOP (M)**	COCO	92	79	31	28	31	28	31	28	62	56	62	56	62	56	78	69
**Top1**		64.34	**60.3**	33.41	31.05	33.54	31.5	33.42	31.17	63.33	61.54	64.21	62.53	61.53	66.05	68.34	64.23
**Top5**		69.23	**64.8**	40.53	37.31	40.83	37.11	41.53	37.41	69.25	65.72	71.63	64.53	66.85	66.55	75.46	72.5

## Data Availability

Data will be made available on request.
